# Viscosity Approximation of PDMS Using Weibull Function

**DOI:** 10.3390/ma14206060

**Published:** 2021-10-14

**Authors:** Andrzej Chmielowiec, Weronika Woś, Justyna Gumieniak

**Affiliations:** The Faculty of Mechanics and Technology, Rzeszow University of Technology, ul. Kwiatkowskiego 4, 37-450 Stalowa Wola, Poland; achmie@prz.edu.pl (A.C.); j.gumieniak@prz.edu.pl (J.G.)

**Keywords:** poly(dimethylosiloxane), damping fluid, viscous damper, TVD, automotive

## Abstract

The viscosity of a fluid is one of its basic physico-chemical properties. The modelling of this property as a function of temperature has been the subject of intensive studies. The knowledge of how viscosity and temperature variation are related is particularly important for applications that use the intrinsic friction of fluids to dissipate energy, for example viscous torsional vibration dampers using high viscosity poly(dimethylsiloxane) as a damping factor. This article presents a new method for approximating the dynamic viscosity of poly(dimethylsiloxane). It is based on the three-parameter Weibull function that far better reflects the relationship between viscosity and temperature compared with the models used so far. Accurate mapping of dynamic viscosity is vitally important from the point of view of the construction of viscous dampers, as it allows for accurate estimation of their efficiency in the energy dissipation process.

## 1. Introduction

**Figure 1:**
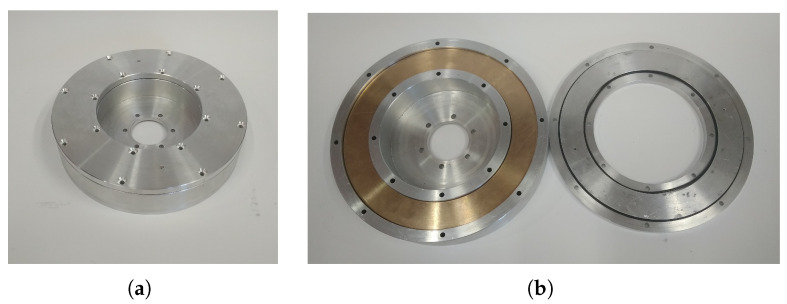
Viscous torsional vibration damper: (**a**) view with the cover closed, (**b**) cross-section.

One of the serious threats to the proper operation of engines and crankshafts is torsional vibrations. They cause faster wear of elements of the crank-piston, timing and drive systems. In order to eliminate vibrations, and thus limit damage to multi-cylinder engines, torsional vibration dampers are used. They were used for the first time at the beginning of the 20th century in the USA to damp vibrations of torsional camshafts in submarine engines [1,2]. Viscous torsional vibration dampers (Figure 1) consist of a housing, a cover, and an inertia ring immersed in the fluid filling the housing. Additionally, the ring is held in position by radial or axial bearings [2,3,4,5,6,7].

A properly selected suppression medium should be non-toxic, show low compatibility, good chemical stability, high flash point, non-flammability, low sensitivity of viscosity to changes in temperature, resistance to cold and aging [7,8].

Polysiloxanes meet the above requirements, and that is why dampers are filled with silicon oils with viscosities of up to 1,000,000 cSt. The most commonly used oil is stabilized poly(dimethylsiloxane) based oil produced by Bayer [9] and Wacker [10]. Depending on the degree of oil contamination and its viscosity, a damper is either approved for further use or regenerated during service. It is therefore important to know the rheological properties of such oil [2,8,11].

Polysiloxanes are organosilicon polymers with the general chemical formula ${\left[R_{2}\mathrm{SiO}\right]}_{n}$. They consist of an alternating silicon-oxygen backbone chain and functional side groups. Being structured this way, they show a number of valuable properties depending on the size of molecules, which distinguish them from organic materials [12,13].

**Figure 2:**
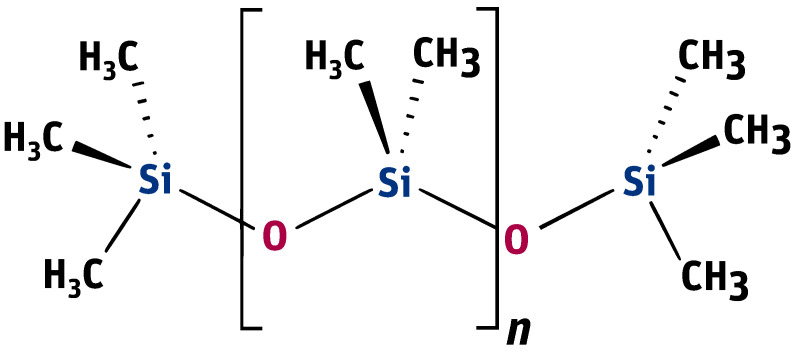
Formula of poly(dimethylsiloxane) containing methyl groups.

Linear PDMS, poly(dimethylsiloxane), containing methyl groups (presented in Figure 2), is the most popular representative of this group of compounds.

Depending on the value of n, which is the number of repeating monomer units, which can vary from 10 to up to 10,000, PDMS has a liquid or semi-solid form for large values of *n*. On the other hand, taking into account the kinematic viscosity of PDMS, there are fluids with low (0.65–20 cSt), medium (50–100 cSt) and high (5000–250,000 cSt) viscosity, and rubbers (over 500,000 cSt) [14,15]. The relationship between the viscosity of poly(dimethylsiloxanes) and molecular weight is presented in Table 1.

The unique chemical structures of poly(dimethylsiloxanes) ensure high chain mobility, which translates into high chemical stability, extremely low glass transition temperature of about −125 ${}^{\circ }C$ and high gas permeability.

Thanks to their high thermal stability, poly(dimethylsiloxanes) can be used in many industries. Among others, they are used as a material for making microsystems, as MEMS precursors (microelectromechanical systems), and microfluidic components [17,18,19]. Properly selected viscosity makes it possible to use PDMS for creating a coating and then separating rubber, plastic or metal castings from molds. In manufacturing and chemical processes, mainly in anhydrous systems, a small addition of poly(dimethylsiloxane) prevents foaming, e.g., in oil production. It is also a perfect additive to loose materials, preventing or reducing their tendency to caking. In its fluid form PDMS demonstrates excellent lubricity on plastic and elastomeric surfaces. Other examples of PDMS application include mechanical shock absorbers in aircraft seats and dashboards, engine sealants, oils, adhesives, as well as thermal and acoustic insulators [14,18,20,21,22].

As already mentioned, the analysis of the silicone oil properties is an extremely important aspect of designing and servicing viscous dampers used to damp the torsional crankshafts vibrations of multi-cylinder internal combustion engines.

Viscosity, or internal friction, is the ability of a fluid to dissipate energy as its molecules move in relative motion. In other words, it is the ability to increase the entropy of a system of particles in a fluid by converting their ordered motion manifested by flowing into disordered motion manifested by rising temperature. From this point of view, this process has many properties that allow it to be modeled as a stochastic process. Therefore, it is reasonable and justified to search for an appropriate approximation of viscosity as a function of temperature among the scalled probability distributions.

Viscosity is one of the major indicators of the quality and performance of a damping medium. It changes along with an increase or a decrease in the shear rate, a change in the operating temperature, and the oil aging process. It is extremely important to determine how viscosity changes depending on temperature. This knowledge allows for simulating the operation of a damper already at the design stage. By precisely determining the viscosity once the damper has reached its operating temperature, it is possible to determine whether the device will be able to dissipate torsional energy quickly enough. Such a simulation also allows conclusions to be drawn about the damper operating temperature and about the risk of it being dangerously exceeded. It is important because overheating of the device may lead to uncontrolled pressure increase and leakage of the housing. It can therefore be concluded that the exact knowledge of changes in viscosity as a function of temperature is crucial for the construction of a viscous vibration damper and its reliable operation [7,11,23,24].

The modelling of the viscosity of fluids has been intensively researched since the end of the 19th century, when in 1886 Reynolds [25] presented the first model of the viscosity of fluids. At the beginning of the 20th century, Vogel [26], Fulcher [27], Tammann i Hasse [28] and Walther [29,30,31] presented their models of this phenomenon. The models developed by the first four authors are general and are still commonly used to this day as VFT or VFTH (the Vogel–Fulcher–Tammann–Hasse viscosity model), for example in the publications of Nascimento and Aparicio [32], Jancewicz et al. [33] and Zhou and Wang [34]. A very good review of the general viscosity models was conducted by Seeton [35], who introduced a new viscosity model for hydrocarbons and hydrofluorocarbons (halocarbones).

The publications on the correlation between viscosity and temperature for fluids present numerous models relating to this relationship. Due to the limitations, the models have been modified over the years. The authors paid special attention to the equation proposed by Fulcher in 1925, due to the analysed material which included silicate [27].

Taking into account the widespread use of siloxane preparations, there is great interest in their stability and degradation paths, especially at various temperatures. Understanding the relationship between the structure and properties of polysiloxanes is important in predicting how they will behave in specific conditions, and thus to determining the efficiency and lifetime of the materials made of them [3,18,36].

The literature provides information on the existing models of viscosity changes resulting from rheological tests performed for poly(dimethylsiloxanes) with relatively low viscosities [37,38]. Such data are also provided by producers, but often in a very narrow temperature range. There is little information about this for PDMS with higher viscosities, possibly because measurements using these polymers pose more difficulties. However, it is a very interesting and useful issue, among others due to the increasing area of application of highly viscous PDMS [39,40].

Among the available information about changes in PDMS viscosity, there is a constitutional equation taking into account the dependence of visco-elasticity on temperature for silicone oil AK 1,000,000 STAB by Wacker Chemie, consisting of the five-element White-Metzner model, derived to reproduce the Weissenberg effect occurring during the rotational measurement carried out by Kőkuti et al. in the Comsol environment [41,42,43].

It should be emphasized that the general models of the viscosity of fluids as a function of temperature are not overly accurate in the case of highly viscous fluids, as is the case for poly(dimethylsiloxanes) used to dampen vibrations. At 25 ${}^{\circ }C$ the kinematic viscosity of these fluids ranges from $10^{4}$ to up to $10^{6}$ cSt. Viscosity tests showed that the Fulcher formula used by the producer to model viscosity significantly differs from actual measurement results. Therefore, an attempt was made to create a mathematical approximation that would reflect the relationship between viscosity and temperature for high viscosity poly(dimethylsiloxane) more accurately. Taking into account the fact that the phenomenon of changes in viscosity as a function of temperature has certain properties of a stochastic process, a decision was made to use the three-parameter Weibull function, which is often used to model various physical phenomena.

The current state of knowledge and the available models describing changes in the viscosity of fluids and poly(dimethylsiloxane) inspired the authors to create a new method that approximates changes in viscosity as a function of temperature for PDMS with viscosities of tens of thousands of cSt. Such a method may be useful, for example, in preventing or controlling the thermal decomposition of elements made of this polymer, and thus in prolonging their proper operation [17,18].

The following sections of the article describe the experimental tests conducted in order to determine the actual dependence of poly(dimethylsiloxane) viscosity on temperature. Next, a universal method of viscosity approximation using the Weibull function is presented. The method was used to create PDMS viscosity formulas for viscous oils with given nominal viscosities. Finally, a one-parameter formula of viscosity for all tested oils is presented.

## 2. Materials and Methods

The tests were performed on poly(dimethylsiloxane) samples with nominal kinematic viscosities of 10,000 cSt, 30,000 cSt and 60,000 cSt respectively, at 25 ${}^{\circ }C$. The tested PDMS was manufactured by Clearco (Clearco Products Co., Inc., 15 York Road, Willow Grove, PA, USA) Products.

Viscosity was measured using a Brookfield (AMETEK Brookfield, 11 Commerce Blvd., Middleboro, MA 02346, USA) DVE rotational viscometer (model RV). The viscosity of every PDMS sample was measured in the range from −20 ${}^{\circ }C$ to 150 ${}^{\circ }C$. Below room temperature values were obtained by cooling the poly(dimethylsiloxane) in a refrigerator. After the temperature stabilized, the PDMS was removed from the refrigerator and measured. The temperature was controlled by placing a thermocouple as close to the spindle as possible. The step between measurement values was not always equal, but was not higher than 5 ${}^{\circ }C$. Each sample was first cooled in a freezer and then placed in a special vessel heated in an oil bath on a heating plate. The measurements were made in accordance with the measuring procedure provided together with the device by the producer. RV06 and RV05 spindles were used to perform the tests.

### 2.1. Inaccuracy of the Exponential Model

The viscosity of poly(dimethylsiloxane) as a function of temperature is the basic physico-chemical property that should be taken into account when designing and modelling a damper. Although a damper works as such after a certain time at a certain temperature, the start-up phase, which lasts up to several hours, involves significant changes in the viscosity of the damping oil. Consequently, it is very important to have a function that can accurately relate dynamic viscosity with temperature.

For the purpose of this article, three oils with nominal viscosities of 10,000 cSt, 30,000 cSt and 60,000 cSt, respectively, at 25 ${}^{\circ }C$ were tested. The catalogues provided by producers specify the basic physico-chemical properties of the offered oils. These include, among others, information such as:

- nominal kinematic viscosity determined at 25 ${}^{\circ }C$:
  (a) $\nu_{25}^{10}=10,000\;\mathrm{cSt}=0.01\;m^{2}\cdot s^{-1}$,
  (b) $\nu_{25}^{30}=30,000\;\mathrm{cSt}=0.03\;m^{2}\cdot s^{-1}$,
  (c) $\nu_{25}^{60}=60,000\;\mathrm{cSt}=0.06\;m^{2}\cdot s^{-1}$
- nominal density at 25 ${}^{\circ }C$: $\rho_{25}=976\;\mathrm{kg}\cdot m^{-3}$,
- relative volumetric thermal expansion coefficient $c=0.00093{\;}^{\circ }C^{-1}$.

**Figure 3:**
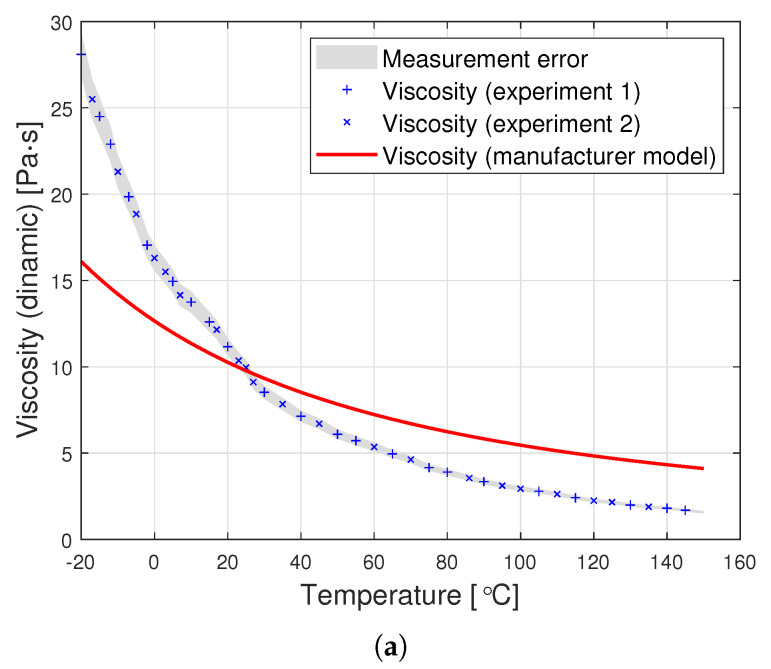

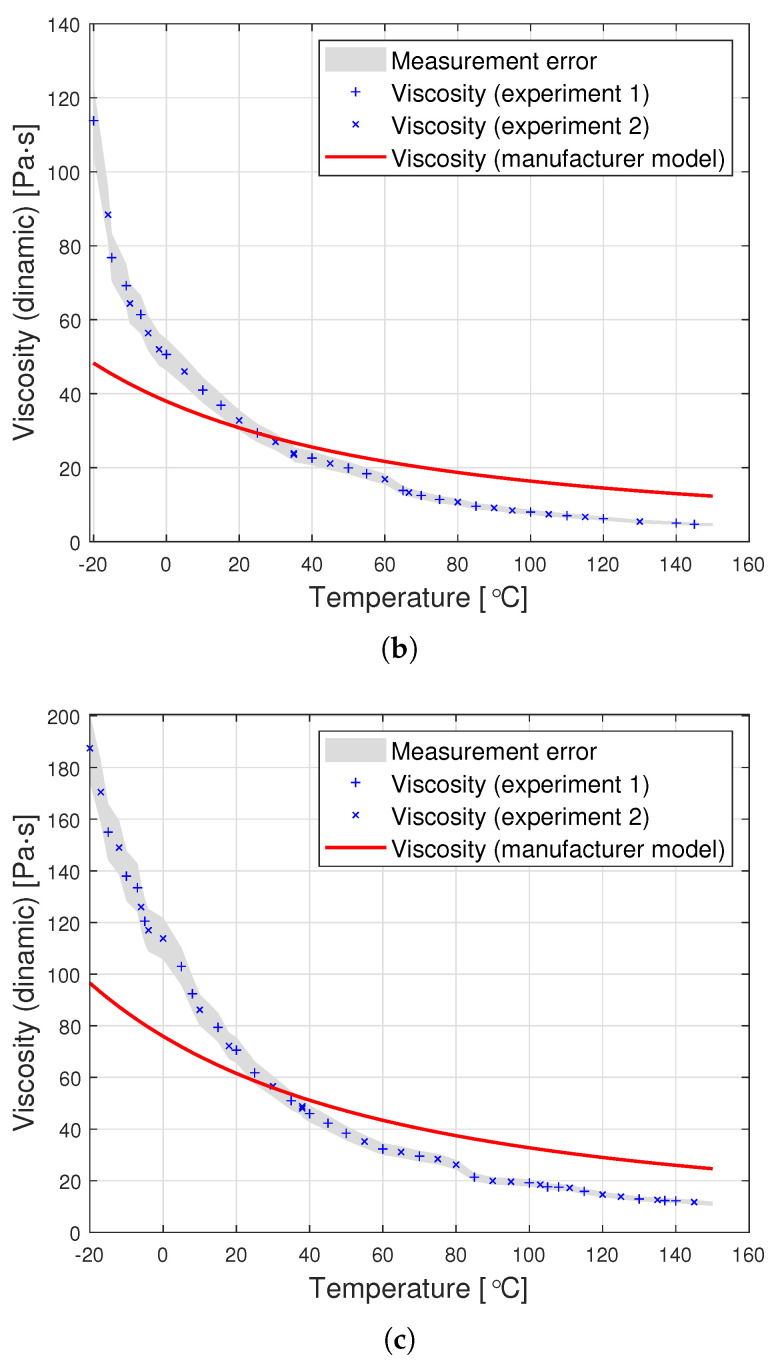
Graphs of PDMS viscosity as a function of temperature for experimental measurements and the producer’s model, for oils with nominal kinematic viscosities: (**a**) 10,000 cSt, (**b**) 30,000 cSt, (**c**) 60,000 cSt.

Dynamic viscosity measurement is carried out by setting a given spindle rotation speed per minute (RPM). The spindle rotated, generating the following shear rate values: from 10 to 100 (0.17 Hz–1.67 Hz) for an oil with a nominal kinematic viscosity of 10,000 cSt, from 5 to 50 (0.08 Hz–0.83 Hz) for 30,000 cSt, and from 2 to 50 (0.03 Hz–0.83 Hz) for 60,000 cSt. According to the data from the Clearco catalog card Properties of Polydimethylsiloxanes from 0.65 cSt to 2.5 million cSt, the tested oils remained a newtonian liquid at these speeds. PDMS in the studied range of shear velocity does not change its rheological properties. Measurements for each viscosity were performed in two independent series. The results from different series of measurements are shown in Figure 3 with different shapes of points. Taking into account the selected measurement method, it was assumed that the error would be estimated as the relative variability of the measurement process. The error was estimated using standard deviation $\sigma =\sqrt{\frac{1}{k-1}\sum_{i=1}^{k}e_{i}^{2}}$, where $e_{i}=\left|\frac{\eta_{i-1}+\eta_{i+1}}{2\eta_{i}}-1\right|$ is the relative measurement error provided that there is a linear relationship between three consecutive measurements. Assuming a normal error distribution, the error value was specified as 3σ.

The function provided by the producer, defining the relationship between kinematic viscosity and temperature, is given by the formula:

(1) $$\begin{array}{c} \nu \left(T\right)=\exp\left(\frac{763.1}{273+T}-2.559+\ln\nu_{25}\right) \end{array}$$

where the temperature *T* is given in ${}^{\circ }C$, and the parameter $\nu_{25}$ should be replaced accordingly with one of the values $\nu_{25}^{10}$, $\nu_{25}^{30}$, or $\nu_{25}^{60}$. It can be noticed that the model used by the producer is in fact an adaptation of the formula presented by Fulcher [27].

The data provided by the producer allow for determining the function defining the relationship between dynamic viscosity and temperature on the basis of the relationship η(T)=ρ(T)ν(T). The formula determining oil density ρ(T) can be obtained on the basis of nominal density $\rho_{25}$ at 25 ${}^{\circ }C$ and the coefficient of relative thermal expansion *c*. Assuming that the coefficient *c* is constant, we get the following formula for PDMS volume as a function of temperature $V\left(T\right)=V_{25}\left(1+c\left(T-25\right)\right)$. Applying the law of conservation of mass, we obtain the relationship $\rho_{25}V_{25}=\rho \left(T\right)V\left(T\right)=\rho \left(T\right)V_{25}\left(1+c\left(T-25\right)\right)$ which leads to the following formula:

$$\begin{array}{c} \rho \left(T\right)=\frac{\rho_{25}}{1+c(T-25)} \end{array}$$

The compliance of the value of the c coefficient and its independence from temperature has been confirmed experimentally by the authors. The oil producer’s final formula for dynamic viscosity as a function of temperature can be expressed as follows:

(2) $$\begin{array}{c} \eta \left(T\right)=\frac{\rho_{25}}{1+c(T-25)}\exp\left(\frac{763.1}{273+T}-2.559+\ln\nu_{25}\right) \end{array}$$

It should be emphasized that the factor ρ(T) should not be taken as a constant due to the relatively high coefficient of relative thermal expansion *c*. In the tested temperature range (from −20 to $150{\;}^{\circ }C$), silicone oil increases its volume by as much as 16%.

The silicone oil producer (Clearco Products) warns that the Formula (1) should be used only for temperatures ranging from 25, to $250{\;}^{\circ }C$. Another manufacturer of silicone oils—Shin Etsu (Shin-Etsu Chemical Co., Ltd., Asahi Seimei Otemachi Bldg., 6-1, Ohtemachi 2-chome, Chiyoda-ku, Tokyo, Japan)—uses exactly the same model in the temperature range from −25 to $250{\;}^{\circ }C$. Unfortunately, testing the oil in such a range was beyond the scope of the apparatus at the disposal of the authors. Perhaps the viscosity model commonly used by silicone oil manufacturers works well for other viscosities. Nevertheless, we find it very problematic to create a single-parameter formula that would model well the viscosity of all oils at the same time. The graphs in Figure 3 present a comparison of the experimental results with the data calculated based on model (2) also for temperatures below $25{\;}^{\circ }C$. This clearly shows how much the adopted model of viscosity as a function of temperature differs from reality. The modification of the Fulcher [27] model used by both manufacturers may be better suited to the experimental values. Unfortunately, such an operation makes it necessary to operate with temperatures below 0K. Such modeling, however, does not make much sense from a physical point of view. Seeton [35] indicates that Walther’s formula as introduced by Barr may be a good model for oils. The authors confronted the experimental results with this model. For the values of a=0.8, A=1 and the viscosity ν given in cSt, the following values of the coefficients *b* and *c* were obtained: $b_{10,000}=271.53,c_{10,000}=0.59,b_{30,000}=266.07,c_{30,000}=0.57,b_{60,000}=178.26$, $c_{60,000}=0.49$. All these models offer a very good fit with the experimental results. Pearson’s correlation coefficient for the Walther’s formula is insignificantly lower than in the case of the approximation presented in this article. Nevertheless, due to the lack of regularity in the values of the *b* coefficient, it is not possible to reduce the model determined by the Walther’s formula to a one-parameter model. These high discrepancies encouraged the authors to try to develop a!new approximation method that would better describe the behaviour of dynamic viscosity as a function of temperature, especially for high nominal kinematic viscosities. A new approach to the problem of poly(dimethylsiloxane) viscosity approximation will be presented later in the article.

### 2.2. Weibull Distribution

At the end of the 1930s, Weibull [44] introduced a new probability distribution, which was used in modelling the breaking force phenomenon. Then, he developed his work and showed how his distribution can be used in a number of other applications [45]. In fact, the first publications dedicated to this distribution were authored by Fréchet [46], and Fisher and Tippett [47]. However, it was Weibull who introduced the scale and position parameter to the distribution, thus making it very important for practical applications.

As a result of the strong interest in statistical methods in engineering, the Weibull distribution has been used in numerous areas of technical and engineering sciences over the last 40 years. The following list is only a small fraction of what can now be found in the literature about the application of the Weibull distribution. Nevertheless, it illustrates well the versatility of this distribution, as well as the fact that to this day it is very often used to model such phenomena as: steel yield point, steel fatigue life [44], glass breaking strength [48], pitting corrosion of pipes [49], adhesion wear of metals [50], failure rate of carbon fibre composites [51], failure rate of coatings [52], failure rate of brittle materials [53], failure rate of composite materials [54], wear of concrete elements [55], fatigue life of aluminium alloys with high entropy [56], fatigue life of Al-Si castings [57], modelling of the power curve of a wind turbine [58], strength of materials using banana fibre [59], strength of polyethylene terephthalate fibres [60], and failure rate of joints under shear [61]. The Weibull distribution is also widely used to model the coalescence process of foams and emulsions, which was very well presented in the review article by Suja et al. [62]. This article will add yet another item to this long list: approximation of the viscosity of poly(dimethylsiloxane) as a function of temperature.

From a physical point of view, viscosity can be interpreted at the molecular level in terms of probability. It depends on the probability of contact between molecules. Therefore, it seems like a good idea to use a probability distribution to approximate the viscosity. From a mathematical point of view the Weibull function was chosen to approximate the viscosity function due to the fact that it is very universal and its formula covers a lot of probability distributions. Of course, with a high degree of probability it is possible to fit almost any exponential function parameterized with three quantities to the experimentally measured viscosity function. The problem arises, however, when we want to indicate a set of parameters suitable for several liquids on the basis of such a match. Very often, fitting for a single function does not give the possibility of extending the model to many functions.

There are several ways of defining the Weibull distribution [63]. One of them is the definition by a probability distribution function expressed as follows:

(3) $$\begin{array}{cc} f(t) & =\frac{\beta }{\alpha }{\left(\frac{t-\tau }{\alpha }\right)}^{\beta -1}\exp\left(-{\left(\frac{t-\tau }{\alpha }\right)}^{\beta }\right) \end{array}$$

where α,β>0,τ≥0,t≥τ, while α is the scale parameter, β is the shape parameter, and τ is the position parameter. Using these designations, the distribution function *F* is determined as:

(4) $$\begin{array}{cc} F(t) & =1-\exp\left(-{\left(\frac{t-\tau }{\alpha }\right)}^{\beta }\right) \end{array}$$

Moreover, the expected value and variance are given by:

$$\begin{array}{cc} \mu & =\tau +\alpha \Gamma \left(1+\frac{1}{\beta }\right)\\ \sigma^{2} & =\alpha^{2}\left[\Gamma \left(1+\frac{2}{\beta }\right)-\Gamma^{2}\left(1+\frac{1}{\beta }\right)\right] \end{array}$$

**Figure 4:**
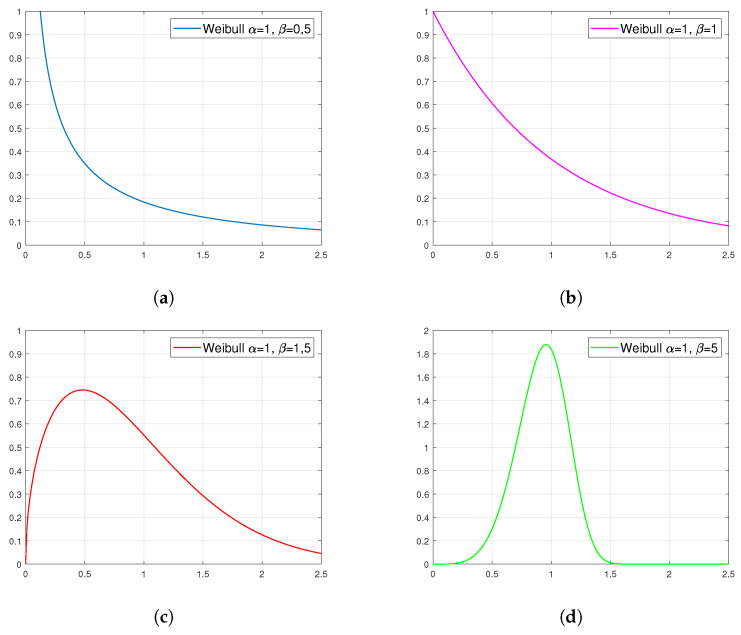
Examples of density plots for the Weibull distribution with specific parameters: (**a**) α=1, β=0.5, (**b**) α=1, β=1, (**c**) α=1, β=1.5 and (**d**) α=1, β=5.

Figure 4 presents examples of plots of the probability density function for the Weibull distribution. They show that the family of these distributions covers a very wide spectrum of cases. In addition, the observance of the shape of these distributions suggests that those presented in figures (a) and (b) can fit well in the approximating of viscosity as a function of temperature.

Moreover, the Weibull distribution can be transformed to linear relationships. Namely, using Equation (4) the following relationship was obtained:

$$\begin{array}{cc} 1-F(t) & =\exp\left(-{\left(\frac{t-\tau }{\alpha }\right)}^{\beta }\right) \end{array}$$

By taking the double log:

$$\begin{array}{cc} \ln(-\ln(1-F(t))) & =\beta \ln(t-\tau )-\beta \ln\alpha \end{array}$$

By determining the value τ and using the following variable replacement:

(5) $$\begin{array}{cc} Y & =\ln(-\ln(1-F(t)))\\ X_{\tau } & =\ln(t-\tau ) \end{array}$$

the family of linear relationships indexed by the parameter τ was obtained

(6) $$\begin{array}{cc} Y & =\beta X_{\tau }-\beta \ln\alpha \end{array}$$

This simple transformation will be used in the new stochastic approach to modelling dynamic viscosity as a function of temperature outlined below.

### 2.3. Approximating of Viscosity as a Function of Temperature Using the Weibull Function

For oils with nominal kinematic viscosities of 10,000 cSt, 30,000 cSt and 60,000 cSt dynamic viscosity was measured at different temperatures. As a result of the experiment for each oil a certain set of results was obtained $\left(T_{i},\eta_{i}\right)$ for i∈{1,2,…,n}. The $\eta_{i}$ values describe the viscosity of the given poly(dimethylsiloxane) measured at the temperature $T_{i}$. Figure 3 presents graphs comparing the model (2) with experimental data. They clearly show that the model provided by the producer differs considerably from the actually measured values. In order to create a more accurate approximation, the Weibull function (3) will be used. The optimal selection of the parameters α, β and τ will be the starting point for developing a general formula for the viscosity of the tested poly(dimethylsiloxanes). It should be stressed that the procedure described below is general and may also be used to approximate the viscosity of other substances. The method of normalizing the viscosity function presented below should be regarded as a purely technical operation. It is carried out in order to introduce a common measure for all viscosities that are subjected to the proposed approximation method.

Since the Weibull function is the density of the probability distribution, its integral is normalized and equals 1. Therefore, the first step is to normalize the viscosity measurement results. The normalizing constant was determined with the following expression

$$G=\left(1+\varepsilon \right)\cdot \sum_{i=1}^{n-1}D_{i}\cdot \frac{\left(\eta_{i}+\eta_{i+1}\right)}{2}$$

where $\eta_{i}$ are the observed viscosities at temperatures $T_{i}$, a $D_{i}=T_{i+1}-T_{i}$ is the difference of temperatures between successive measurements. Parameter ε is any positive constant. It determines the scale of the error made when considering the dynamic viscosity function in a closed temperature range, compared to the theoretical range from minus to plus infinity. In this article it was assumed that ε=0.01. Then, the normalized values are determined

(7) $$F_{i}=\sum_{k=1}^{i}D_{k}\cdot \frac{\eta_{k}+\eta_{k+1}}{2G}$$

for i=1,2,…,n−1, expected to reflect the shape of the cumulative distribution function of a certain Weibull distribution with unknown parameters. Next, the following calculation are made:

$$\begin{array}{c} d=\max\{D_{i}:i=1,2,\ldots ,n-1\} \end{array}$$

The family $X_{\tau }$ described by Formula (5) is limited to the value $\tau \in [T_{1}-d,T_{1})$. In the discussed case, for all tested oils $T_{1}=-20{\;}^{\circ }C$, and $d=5{\;}^{\circ }C$, therefore $\tau \in [-25{\;}^{\circ }C,-20{\;}^{\circ }C)$. In order to optimally adjust the parameters to the experimental measurement results, a grid of points was set $\Omega =\{\tau_{1},\tau_{2},\ldots ,\tau_{m}\}$, where $\tau_{i}\in \left[T_{1}-d,T_{1}\right)$ from which the parameter τ will be selected that most accurately reflects the viscosity distribution as a function of temperature.

Next, for all values $\tau_{k}\in \Omega$ (k=1,2,…,m) and for i=1,2,…,n−1 the value matrix should be determined:

$$\begin{array}{c} X_{ki}=\ln\left(T_{i}-\tau_{k}\right) \end{array}$$

Moreover, there is created a vector:

$$\begin{array}{c} Y_{i}=\ln\left(-\ln\left(1-F_{i}\right)\right) \end{array}$$

for i=1,2,…,n−1, where $F_{i}$ is calculated using Formula (7).

Our task is to determine such parameters α,β and τ, for which the Weibull distribution given by Equation (3) will accurately represent the shape of the viscosity function η(T). In other words, the goal is to find function f(T;α,β,τ), that after applying a certain linear transformation A·f(T)+B will be an approximation of function η(T).

Note that each row of the matrix $X_{ki}$ is linearly related to the vector $Y_{i}$ in accordance with Formula (6). Consequently, for all k=1,2,…,m it is possible to determine the linear regression coefficients $a_{k}$, $b_{k}$ for the set of pairs $\left\{\left(X_{ki},Y_{i}\right)\right\}$ and use them as the basis for determining the parameters of the distribution:

$$\begin{array}{c} \alpha_{k}=\exp\left(-\frac{b_{k}}{a_{k}}\right),\;\;\;\;\;\;\;\;\beta_{k}=a_{k} \end{array}$$

Next, for all k=1,2,…,m and i=1,2,…,n−1 the matrix of values is determined

$$\begin{array}{c} f_{ki}=\frac{\beta_{k}}{\alpha_{k}}{\left(\frac{T_{i}-\tau_{k}}{\alpha_{k}}\right)}^{\beta_{k}-1}\exp\left(-{\left(\frac{T_{i}-\tau_{k}}{\alpha_{k}}\right)}^{\beta_{k}}\right) \end{array}$$

Then, for k=1,2,…,m the linear regression coefficients $A_{k}$, $B_{k}$ were calculated for the set of pairs $\left\{\left(\eta_{i},f_{ki}\right)\right\}$ and define the matrix of values estimating viscosity as:

$$\begin{array}{cc} {\hat{\eta }}_{ki} & =A_{k}\cdot f_{ki}+B_{k} \end{array}$$

for k=1,2,…,m, and i=1,2,…,n−1. From the matrix defined this way *k* will be chosen, for which the vector ${\hat{\eta }}_{k}=\left({\hat{\eta }}_{k1},{\hat{\eta }}_{k2},\ldots ,{\hat{\eta }}_{k,n-1}\right)$ will be as close as possible to the vector of measurements $\eta =(\eta_{1},\eta_{2},\ldots ,\eta_{n-1})$. One of the following two values was adopted as the meaure of vector fit:

1. residual standard deviation
  $$\begin{array}{c} S_{k}=\sqrt{\frac{\sum_{i=1}^{n-1}{\left(\eta_{i}-{\hat{\eta }}_{ki}\right)}^{2}}{n-3}} \end{array}$$
2. Pearson correlation coefficient
  $$\begin{array}{c} R_{k}=\frac{\sum_{i=1}^{n-1}\left(\eta_{i}-\bar{\eta }\right)\left({\hat{\eta }}_{ki}-\bar{{\hat{\eta }}_{k}}\right)}{\sqrt{\sum_{i=1}^{n-1}{\left(\eta_{i}-\bar{\eta }\right)}^{2}}\sqrt{\sum_{i=1}^{n-1}{\left({\hat{\eta }}_{ki}-\bar{{\hat{\eta }}_{k}}\right)}^{2}}} \end{array}$$

where $\bar{\eta }=\frac{1}{n-1}\sum_{i=1}^{n-1}\eta_{i}$, $\bar{{\hat{\eta }}_{k}}=\frac{1}{n-1}\sum_{i=1}^{n-1}{\hat{\eta }}_{ki}$ dla k=1,2,…,m.

From all the determined estimators ${\hat{\eta }}_{k}$ the one was selected, for which the residual standard deviation $S_{k}$ is the lowest or the one for which the Pearson correlation coefficient $R_{k}$ is the highest. The exact procedure of determining the parameters of the viscosity estimator is presented in Algorithm 1, which operates on the basis of the criterion of minimizing the residual standard deviation. Note that it is also possible to implement the proposed algorithm based on the Pearson correlation coefficient or another criterion.

Determination of the optimal *k* also determines the set of parameters α, β, τ, *A* and *B*, for which the vector of the estimator best fits the vector of real measurements. As a result of the aforesaid procedure carried out for all the tested oils, the parameters $\alpha_{10}$, $\beta_{10}$, $\tau_{10}$, $A_{10}$, $B_{10}$ (PDMS with a nominal viscosity of 10,000 cSt), $\alpha_{30}$, $\beta_{30}$, $\tau_{30}$, $A_{30}$, $B_{30}$ (PDMS with a nominal viscosity of 30,000 cSt) $\alpha_{60}$, $\beta_{60}$, $\tau_{60}$, $A_{60}$, $B_{60}$ (PDMS with a nominal viscosity of 60,000 cSt) were obtained. They form the basis for determining the following values: α, β, τ, *A*, *B* for a unified approximation. For this purpose, the following formulas will be used:

(8) $$\begin{array}{cc} \alpha & =\frac{1}{3}\left(\alpha_{10}+\alpha_{30}+\alpha_{60}\right) \end{array}$$

(9) $$\begin{array}{cc} \beta & =\frac{1}{3}\left(\beta_{10}+\beta_{30}+\beta_{60}\right) \end{array}$$

(10) $$\begin{array}{cc} \tau & =\frac{1}{3}\left(\tau_{10}+\tau_{30}+\tau_{60}\right) \end{array}$$

(11) $$\begin{array}{cc} A & =\frac{1}{3}\left(\frac{A_{10}}{\nu_{25}^{10}}+\frac{A_{30}}{\nu_{25}^{30}}+\frac{A_{60}}{\nu_{25}^{60}}\right) \end{array}$$

(12) $$\begin{array}{cc} B & =\frac{1}{3}\left(\frac{B_{10}}{\nu_{25}^{10}}+\frac{B_{30}}{\nu_{25}^{30}}+\frac{B_{60}}{\nu_{25}^{60}}\right) \end{array}$$

**Algorithm 1:** Procedure of determining the parameters of the viscosity approximation for one substance.
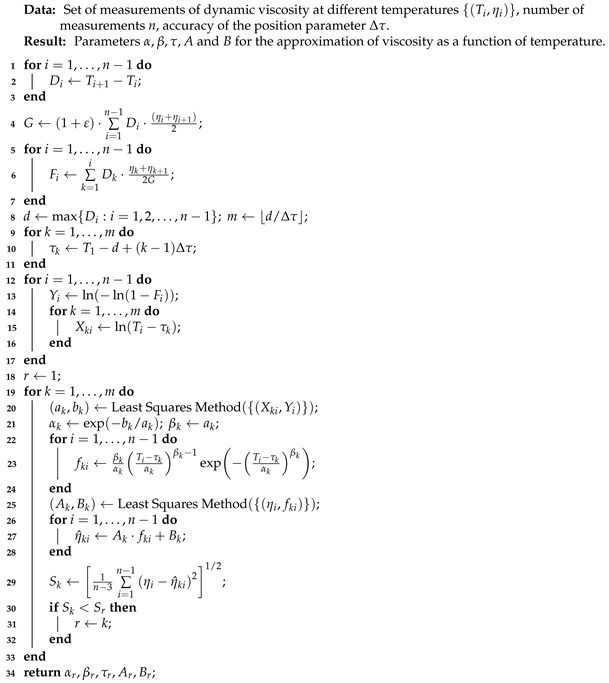


For coefficients defined in this way, a one-parameter approximation of viscosity as a function of temperature is defined:

$$\begin{array}{c} \hat{\eta }\left(T,\nu_{25}\right)=\nu_{25}\left(A\frac{\beta }{\alpha }L{\left(T\right)}^{\beta -1}\exp\left(-L{\left(T\right)}^{\beta }\right)+B\right) \end{array}$$

where $L\left(T\right)=\frac{T-\tau }{\alpha }$, and $\nu_{25}$ is the nominal kinematic viscosity of PDMS at $25{\;}^{\circ }C$.

Moreover, in order to check the accuracy of the proposed approximation and compare the scale of errors with the producer’s model, the coefficient of residual variation *V* will be calculated. This coefficient reflects the percentage level of approximation error. The closer it is to 0, the better the fit of the approximation to empirical data. This value is expressed by the formula

$$\begin{array}{c} V=\frac{S\cdot 100\%}{\bar{\eta }} \end{array}$$

where *S* is the residual standard deviation discussed above. $\bar{\eta }$ is the average of the empirical values of the dynamic viscosity of a given oil.

## 3. Results

**Figure 5:**
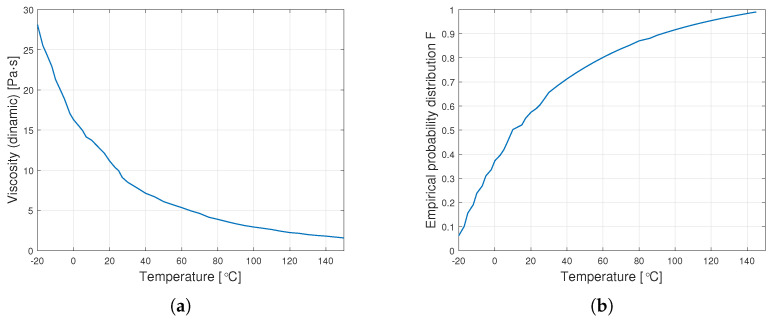
Transition from data on dynamic viscosity to the distribution function (**a**) graph of empirical dynamic viscosity for an oil with a nominal kinematic viscosity of 10,000 cSt, (**b**) empirical distribution function.

Figure 5 shows sample diagrams of the transition from empirical data to the distribution function.

**Figure 6:**
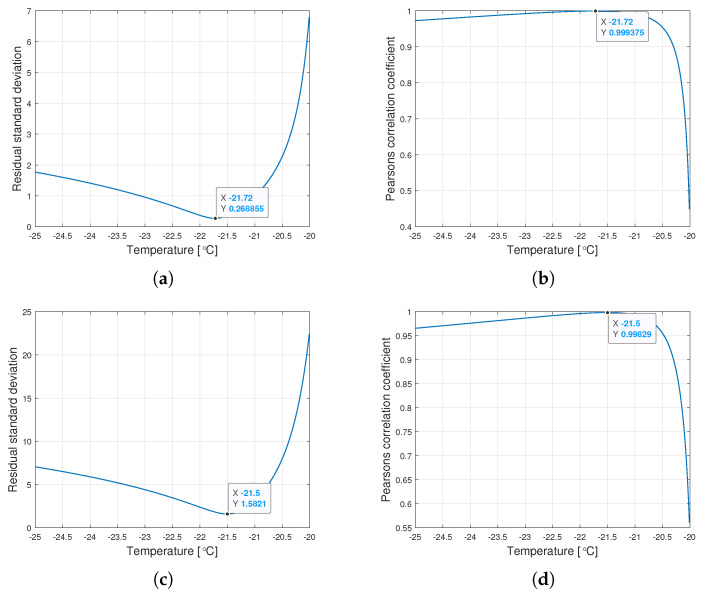

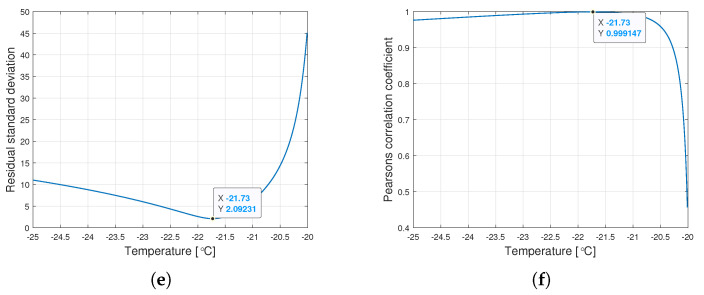
Graphs of the residual standard deviation and the Pearson correlation coefficient for oils of nominal kinematic viscosities: (**a**,**b**) 10,000 cSt, (**c**,**d**) 30,000 cSt, (**e**,**f**) 60,000 cSt.

Interestingly, the minimum value of $S_{k}$ and the maximum value of $R_{k}$ are observed for the same $\tau_{k}$ for a given type of oil. Figure 6 shows the graphs of changes in both coefficients for the tested range of variation of the parameter τ.

The procedure given in Algorithm 1 allows for determining such $\tau_{k}\in \Omega$, for which the Weibull approximation fits the empirical data best. The values $\alpha_{k}$, $\beta_{k}$, $A_{k}$ and $B_{k}$ are connected with the $\tau_{k}$ value determined this way. Table 2 presents the values of the Weibull distribution parameters for which the best approximation of the empirical data have been achieved.

**Figure 7:**
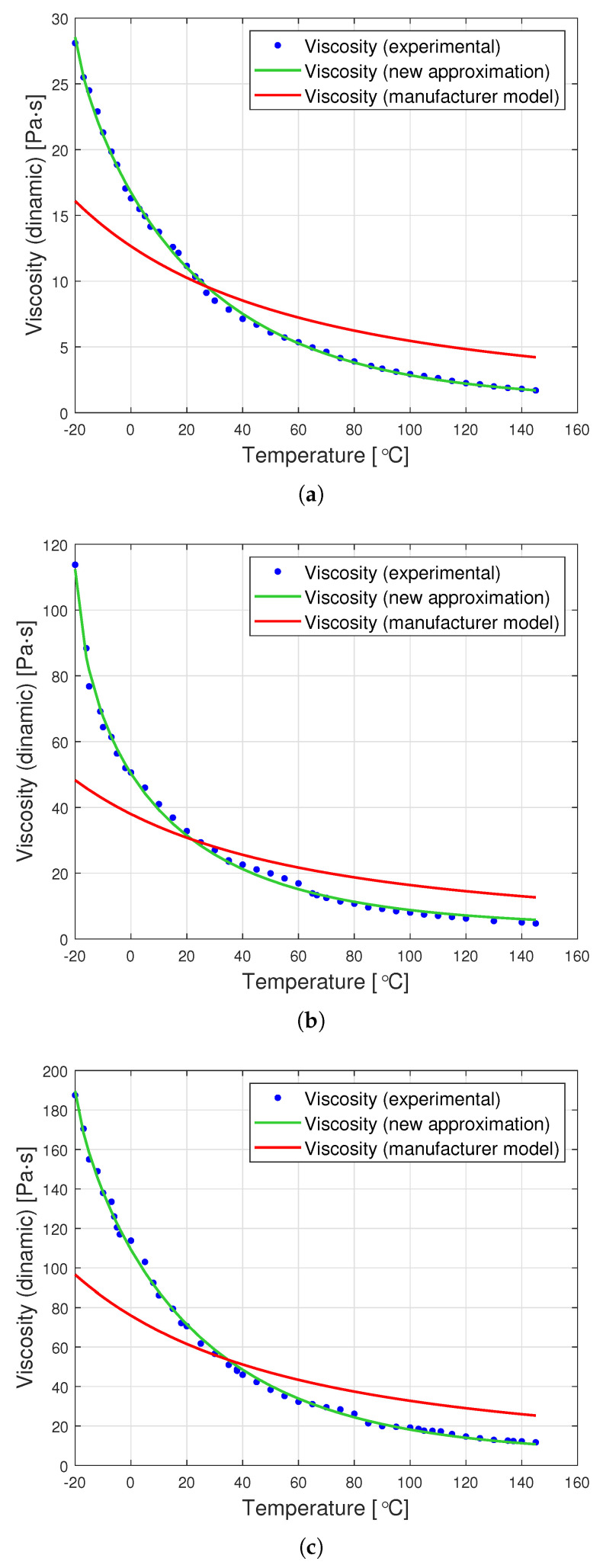
Graphs of dynamic viscosity for oils with nominal kinematic viscosities: (**a**) 10,000 cSt, (**b**) 30,000 cSt, (**c**) 60,000 cSt.

Figure 7 presents graphs showing the dynamic viscosity of the tested oils, the producer’s model (based on the Fulcher model) and the new approximation proposed in this article. The new approximation is presented based on a set of optimal parameters from Table 2. The determined parameters can be used to derive the following analytical formulas for the viscosity of the tested oils as a function of temperature:

- for oil with a nominal kinematic viscosity of 10,000 cSt
  $$\begin{array}{cc} {\hat{\eta }}_{i} & =1209.88\frac{0.95}{46.55}{\left(\frac{T_{i}+21.72}{46.55}\right)}^{0.95-1}\exp\left(-{\left(\frac{T_{i}+21.72}{46.55}\right)}^{0.95}\right)+0.91\\ \; & =24.79{\left(\frac{T_{i}+21.72}{46.55}\right)}^{-0.05}\exp\left(-{\left(\frac{T_{i}+21.72}{46.55}\right)}^{0.95}\right)+0.91 \end{array}$$
- for oil with a nominal kinematic viscosity of 30,000 cSt
  $$\begin{array}{cc} {\hat{\eta }}_{i} & =3608.27\frac{0.87}{41.97}{\left(\frac{T_{i}+21.50}{41.97}\right)}^{0.87-1}\exp\left(-{\left(\frac{T_{i}+21.50}{41.97}\right)}^{0.87}\right)+3.56\\ \; & =74.93{\left(\frac{T_{i}+21.50}{41.97}\right)}^{-0.13}\exp\left(-{\left(\frac{T_{i}+21.50}{41.97}\right)}^{0.87}\right)+3.56 \end{array}$$
- for oil with a nominal kinematic viscosity of 60,000 cSt
  $$\begin{array}{cc} {\hat{\eta }}_{i} & =7919.62\frac{0.95}{46.24}{\left(\frac{T_{i}+21.73}{46.24}\right)}^{0.95-1}\exp\left(-{\left(\frac{T_{i}+21.73}{46.24}\right)}^{0,95}\right)+5.54\\ \; & =162.47{\left(\frac{T_{i}+21.73}{46.24}\right)}^{-0.05}\exp\left(-{\left(\frac{T_{i}+21.73}{46.24}\right)}^{0.95}\right)+5.54 \end{array}$$

The graphs in Figure 7 clearly show that the proposed analytical approximation fits the empirical data very well. On the other hand, the producer’s model based on the Fulcher formula differs significantly from the empirical data. The most significant differences are observed at low temperatures ranging from $-20{\;}^{\circ }C$ to approximately $0{\;}^{\circ }C$.

The goodness of fit of the approximation method presented in the article is particularly apparent when the value of the residual variation coefficient is compared. This is perfectly illustrated in Table 3, presenting a comparison of this coefficient for the producer’s model and for the proposed approximation method. It turns out that the percentage deviation of errors for the proposed new viscosity approximation is up to 15 times smaller than that for the producer’s model.

The analysis of Table 2 shows that the values of the optimal parameters α, β and τ are very similar. This allows the conclusion that by calculating the average of these values, a universal approximation can be obtained for all tested oils. Therefore, the parameters α, β and τ are determined using Formulas (8)–(10)

$$\begin{array}{cc} \alpha & =\frac{1}{3}\left(\alpha_{10}+\alpha_{30}+\alpha_{60}\right)=44.92\\ \beta & =\frac{1}{3}\left(\beta_{10}+\beta_{30}+\beta_{60}\right)=0.92\\ \tau & =\frac{1}{3}\left(\tau_{10}+\tau_{30}+\tau_{60}\right)=-21.65 \end{array}$$

Thus, approximation $\hat{\eta }\left(T;\nu_{25}\right)$ is searched such that for the Weibull formula

$$\begin{array}{c} f_{i}=\frac{0.92}{44.92}{\left(\frac{T_{i}+21.65}{44.92}\right)}^{0.92-1}\exp\left(-{\left(\frac{T_{i}+21.65}{44.92}\right)}^{0.92}\right) \end{array}$$

there is a linear relationship taking into account the nominal kinematic viscosity as a parameter:

$$\begin{array}{cc} {\hat{\eta }}_{i} & =\nu_{25}\;\left(A\cdot f_{i}+B\right) \end{array}$$

Now, *A* and *B* are determined as weighted values using the coefficients from Table 2 in accordance with Formulas (11) oraz (12):

$$\begin{array}{cc} A & =\frac{1}{3}\left(\frac{A_{10}}{\nu_{25}^{10}}+\frac{A_{30}}{\nu_{25}^{30}}+\frac{A_{60}}{\nu_{25}^{60}}\right)=124,419.14\\ B & =\frac{1}{3}\left(\frac{B_{10}}{\nu_{25}^{10}}+\frac{B_{30}}{\nu_{25}^{30}}+\frac{B_{60}}{\nu_{25}^{60}}\right)=100.56 \end{array}$$

where $A_{10}$, $A_{30}$, $A_{60}$ are values from column five of Table 2, while $B_{10}$, $B_{30}$ and $B_{60}$ are values from column six. Then, one common approximation is given by the formula:

(13) $$\begin{array}{cc} {\hat{\eta }}_{i}\left(T_{i};\nu_{25}\right)\; & =\nu_{25}\left(2561.34\;{\left(\frac{T_{i}+21.65}{44.92}\right)}^{-0.08}\exp\left(-{\left(\frac{T_{i}+21.65}{44.92}\right)}^{0.92}\right)+100.56\right)\\ \; & =\nu_{25}\left(2561.34\;L{\left(T_{i}\right)}^{-0.08}\exp\left(-L{\left(T_{i}\right)}^{0.92}\right)+100.56\right) \end{array}$$

where $L\left(T_{i}\right)=\frac{T_{i}+21.64}{44.92}$ and $\nu_{25}$ is replaced with the nominal kinematic viscosity of oil at $25{\;}^{\circ }C$ in units compliant with the SI system, i.e., in $\left[m^{2}\cdot s^{-1}\right]$.

Table 3 shows the values of the coefficient of residual variation *V* calculated for both the producer’s model and for the proposed approximation method. The last column shows the ratio *p* of the residual variation coefficient determined for the producer’s model (from column two) to this coefficient determined for the approximation method proposed in the article (from column three).

**Figure 8:**
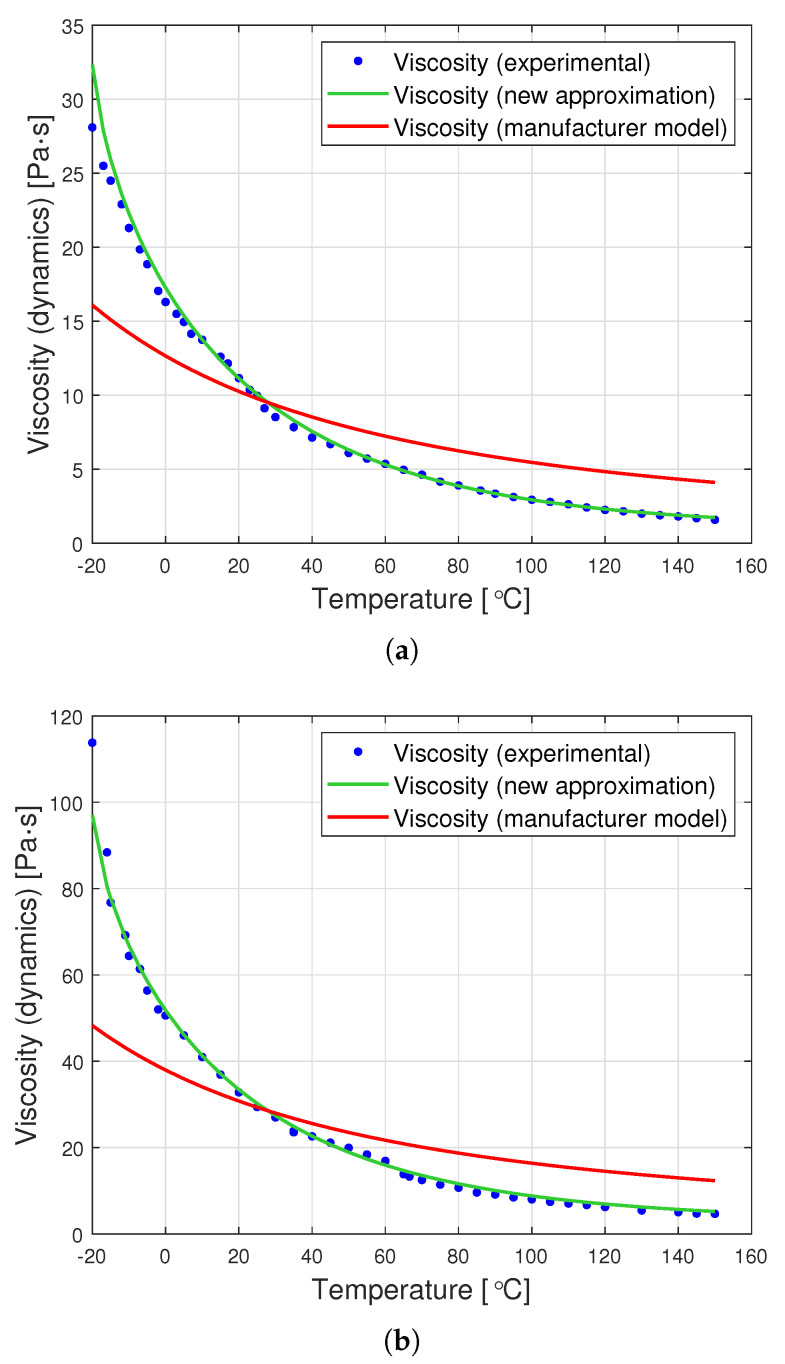

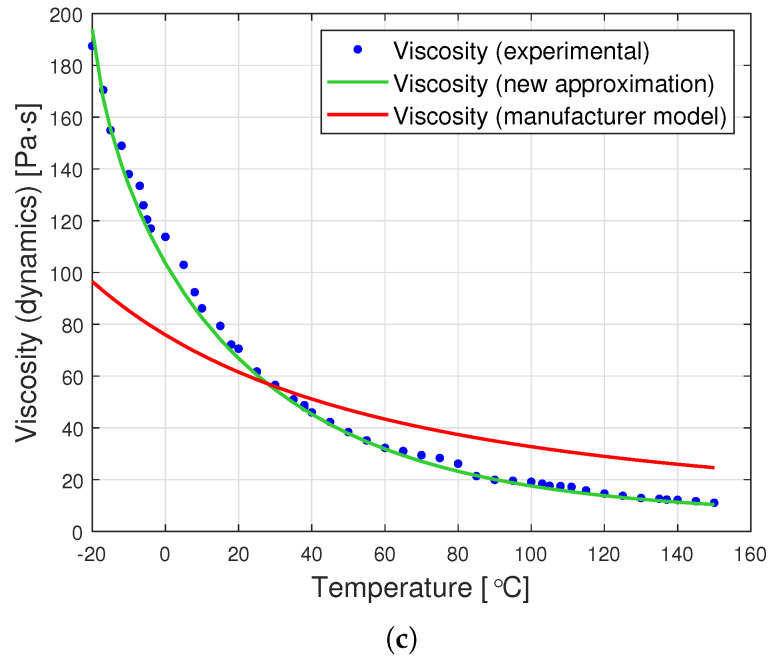
Graphs of dynamic viscosity for oils with nominal kinematic viscosities: (**a**) 10,000 cSt, (**b**) 30,000 cSt, (**c**) 60,000 cSt. (universal approximation).

The graphs in Figure 8 are a graphic representation of the approximation (13). They show the results of the experimental measurement of the dynamic viscosity of the tested oils, the producer’s model, and the proposed joint approximation method (13). It is clearly visible that also the proposed approximation demonstrates a very good fit to the empirical data.

Table 4 presents the values of the coefficient of residual variation for approximation Formula (13). The last column of Table 4 shows the ratio p of the coefficient of residual variation determined for the producer’s model (from column two) to this coefficient determined for the approximation method proposed in the article (from column three).

A comparison of the data from Table 4 with those contained in Table 3 demonstrates that the proposed universal approximation method shows a very good fit to the data. Although this fit is not as high as in the case of dedicated approximations, the use of Formula (13) gives up to eight times smaller errors than the application of the formula proposed by the producer (2).

In view of the above, the authors believe that for PDMS oils with kinematic viscosities ranging from $10^{4}$ to $10^{5}$ cSt and for the temperature range $\left[-20{\;}^{\circ }C,150{\;}^{\circ }C\right]$, it is possible to propose the following one-parameter approximation of dynamic viscosity as a function of temperature $\hat{\eta }\left(T;\nu_{25}\right)$:

$$\begin{array}{cc} \hat{\eta }\left(T;\nu_{25}\right)= & \nu_{25}\left(2561.34\cdot L{\left(T\right)}^{-0.08}\exp\left(-L{\left(T\right)}^{0.92}\right)+100.56\right), \end{array}$$

where $L\left(T\right)=\frac{T+21.64}{44.92}$ and $\nu_{25}$ is replaced with the nominal kinematic viscosity of the tested oil at 25 ${}^{\circ }C$ in units compliant with the SI system, i.e., in $\left[m^{2}\cdot s^{-1}\right]$.

## 4. Conclusions

The article proposes a new one-parameter approximation method of viscosity as a function of temperature for poly(dimethylsiloxanes) with high kinematic viscosities. The approximation formula has been optimized so that it could be used for PDMS with nominal kinematic viscosities ranging from 10,000 to 60,000 cSt and for the temperature range $\left[-20{\;}^{\circ }C,150{\;}^{\circ }C\right]$. Viscosity and temperature ranges selected by the authors is closely related to the applications of silicone oil in viscous torsional vibration dampers for medium-sized diesel engines. The proposed approximation of viscosity will allow for numerical simulations facilitating the damper design process.

It should be emphasized that the presented approximation method is general and may also be used for many other substances, as well as for other temperature ranges. In the case of PDMS, tests extending the lower temperature range for which viscosity will approach infinity seem to be particularly interesting.

## Figures and Tables

**Table 1:** Relationship between PDMS viscosity and molecular weight [16].

PDMS Viscosity [cSt]	Average Number of D-Units ${}^{1}$	Number-Average Molecular Weights
10	15	1300
100	75	5000
1000	200	15,000
10,000	500	37,000
100,000	1000	74,000

${}^{1}$ D refers to the PDMS backbone units consisting of a silicon atom bound to two oxygen atoms and two methyl groups.

**Table 2:** Optimal coefficients of the Weibull distribution.

Nominal Kinematic Viscosity of Oil	α	β	τ	*A*	*B*
10,000 cSt	46.55	0.95	−21.72	1209.88	0.91
30,000 cSt	41.97	0.87	−21.50	3608.27	3.56
60,000 cSt	46.24	0.95	−21.73	7919.62	5.54

**Table 3:** Values of the coefficient of residual variation *V*.

Nominal Kinematic Viscosity of Oil	*V* Determined for the Producer’s Model	*V* Determined for the Proposed Approximation Method	*p*
10,000 cSt	42.92%	2.78%	15.43
30,000 cSt	57.49%	5.31%	10.83
60,000 cSt	50.69%	3.50%	14.47

**Table 4:** Values of the coefficient of residual variation *V* (universal approximation).

Nominal Kinematic Viscosity of Oil	*V* Determined for the Producer’s Model	*V* Determined for the Proposed Universal Approximation Method	*p*
10,000 cSt	42.92%	9.26%	4.64
30,000 cSt	57.49%	11.02%	5.22
60,000 cSt	50.69%	6.54%	7.75

## Data Availability

Data available on request.
